# Rate of rebound in follicle growth after cessation of ovarian stimulation in initial non‐responders: a prospective cohort study

**DOI:** 10.1186/s13048-021-00765-5

**Published:** 2021-01-09

**Authors:** Norbert Gleicher, Andrea Weghofer, Sarah K. Darmon, David H. Barad

**Affiliations:** 1 The Center for Human Reproduction, 10021 New York, NY USA; 2 Foundation for Reproductive Medicine, 10021 New York, NY USA; 3 Stem Cell Biology and Molecular Embryology Laboratory, The Rockefeller University, 10016 New York, NY USA; 4 Department of Obstetrics and Gynecology, Vienna University of Medicine, 1090 Vienna, Austria

**Keywords:** In vitro fertilization (IVF), Low ovarian reserve, Poor prognosis, Failure to respond, gonadotropin isotype, Glycosylation

## Abstract

Previously anecdotally observed rebounds in follicle growth after interruption of exogenous gonadotropins in absolute non-responders were the impetus for here reported study. In a prospective cohort study, we investigated 49 consecutive patients, absolutely unresponsive to maximal exogenous gonadotropin stimulation, for a so-called rebound response to ovarian stimulation. A rebound response was defined as follicle growth following complete withdrawal of exogenous gonadotropin stimulation after complete failure to respond to maximal gonadotropin stimulation over up to 5–7 days. Median age of study patients was 40.5 ± 5.1 years (range 23–52). Women with and without rebound did not differ significantly (40.0 ± 6.0 vs. 41.0 ± 7.0 years, *P* = 0.41), with 24 (49.0%) recording a rebound and 25 (51.0%) not. Among the former, 21 (87.5%) reached retrieval of 1–3 oocytes and 15 (30.6%) reached embryo transfer. A successful rebound in almost half of prior non-responders was an unsuspected response rate, as was retrieval of 1–3 oocytes in over half of rebounding patients. Attempting rebounds may, thus, represent another incremental step in very poor prognosis patients before giving up on utilization of autologous oocytes. Here presented findings support further investigations into the underlying physiology leading to such an unexpectedly high rebound rate.

Whether in association with advanced female age and/or low functional ovarian reserve (LFOR), complete failure to respond to maximal ovarian hyperstimulation with gonadotropins in poor prognosis patients is a common. As a fertility center that serves a disproportional number of highly unfavorable-prognosis patients, the Center for Human Reproduction (CHR) has, therefore, been observing such failures rather frequently. Several years ago, we for the first time anecdotally noticed that some women with complete failure to respond. following short-term interruption in exogenous gonadotropin stimulation, exhibited spontaneously follicle growth and rises in estradiol. When, at those moments, exogenous gonadotropin stimulation was reinstated, follicles responded, and cycles often reached egg retrieval.

We, therefore, in this manuscript describe under the term “rebound” the practice of completely interrupting in women with absolute failure to respond to maximal dosage of exogenous gonadotropin stimulation (i.e., absence of even a single growing follicle and absence of rising estradiol titers) all gonadotropin stimulation for 3–5 days. Patients are then reevaluated, and a rebound is considered successful if by that date at least one growing follicle is detected.

Though our anecdotal impression at that point was that these rebounds in non-responders occurred only rarely and sporadically, rebound attempts became common practice at our center. We, however, more recently decided to investigate this rebound phenomenon in more detail in order to understand its clinical utility and, possibly, inform ourselves in advance as to who will and will not experience such rebounds. Here reported prospective study summarizes our, at times, very surprising findings.

## Materials and methods

### Patient population

The study population comprised 49 consecutive women who presented to the Center for Human Reproduction (CHR) in New York City. Their median age was 40.5 ± 5.1 years (range of 23–52). Our center routinely treats women of very advance ages, though we have not yet achieved IVF pregnancies and live births with autologous oocytes beyond female age 48 [1].

Before presenting to CHR, all patients had received fertility treatments elsewhere, including between 1 and 12 IVF cycles (mean 3.4 ± 3.9). As expected in women with complete absence of response to ovarian stimulation with maximal gonadotropin dosages, demonstrated demonstrated low functional reserve (LFOR), defined as abnormally high age-specific follicles stimulating hormone (FSH) [2] and/or abnormally-low age-specific anti-Müllerian hormone (AMH) [3], with maximal median FSH levels ranging between 20.9 ±. 20.8 mIU/mL to 40.0 ± 7.0 mIU/mL and median AMH between 0.1 ± 0.2 ng/mL and 0.0 ± 0.1 ng/mL, respectively, demonstrating the severity of ovarian dysfunction in reported patients (Table 1).

Here presented data involve first IVF cycles at CHR, though a limited experience with repeat rebounds in same patients is documented separately in the manuscript.

### Initial ovarian preparation and stimulation

Because of LFOR and/or advanced female age, all patients upon presentation were supplemented with dehydroepiandrosterone (DHEA, 25 mg TID, Fertinatal,® Fertility Nutraceuticals, New York, N.Y.) and Coenzyme Q10 (CoQ10, 333 mg TID, Ovoenergen,® Fertility Nutraceuticals LLC, New York, N.Y.) for at least 6 weeks. A first ovarian hyperstimulation was attempted with cycle start on day-2 of menses and involved ovarian stimulation with 450 IU of an FSH product and 150 IU of a human menopausal gonadotropin (hMG) product, with manufacturer choice left to patient and/or insurance mandate. Since older women and/or patients with low functional ovarian reserve (LFOR) at CHR routinely undergo Highly Individualized Egg Retrieval (HIER) [4] and, therefore, have earlier retrievals than standard IVF cycles, these patients do not require preventative treatments for premature spontaneous ovulation with either gonadotropin releasing hormone agonist or antagonist.

### Eligibility

A patient became eligible for this study if after 5–7 days of ovarian stimulation not a single growing follicle beyond antral follicle size was visible on vaginal ultrasound and estradiol (E_2_) had not significantly increased from baseline. Day-7 of stimulation was chosen as cut-off because observations preceding initiation of this study determined that stimulation beyond day-7 never yielded a follicular response in women with here described ovarian characteristics. Patients were then withdrawn from ovarian stimulation with exogenous gonadotropins for 4–6 days (though DHEA and CoQ10 supplementation continued) before undergoing again a repeat vaginal ultrasound and E_2_ evaluation.

If at revaluation at least one growing follicle was visible on vaginal ultrasound and E_2_ had risen from baseline, the patient returned 2 days later for repeat vaginal ultrasound and E_2_ determination. To consider E_2_ to be rising, values had to have increased by at least 30% from prior baseline and one or more follicles had to have increased in size. Once a patient in this way was considered a positive rebound response, ovarian stimulation was reinstituted with 225–300 IU of hMG (please advance to the discussion section for an explanation of dosing). If no response was observed, she was considered a non-responder, her rebound attempt was abandoned and she was advised that further routine exogenous ovarian stimulation was, likely, futile.

### Secondary stimulation in patients with rebound

Responders were stimulated until their lead follicles reached the by HIER predetermined size [4], which could be anywhere between < 12 mm and 18 mm. Most patients were triggered at 12–16 mm lead follicle size. Trigger was routinely 10,000 IU of human chorionic gonadotropin (hCG, either Novarel®, Ferring Pharmaceuticals, Inc., Parsippany, NJ or Pregnyl®, Organon USA Inc., Roseland, NJ). Egg retrieval then took place in routine fashion ca. 34 hours later.

### Statistics

Statistical analyses were performed by CHR’s statistician (SKD). Since distributions were mostly non-parametric (see Appendix), non-parametric statistics were utilized to compare patients who did and did not rebound. Statistical significance was defined as *P* < 0.05. The Wilcoxon-Mann-Whitney test was used for all variables. All statistical analyses were preformed using SAS version 9.4 software.

### Institutional Review Board

As rebounds has been routinely utilized at CHR for years, this study did not change clinical practice. Since data analyses, in addition, were performed using the CHR’s anonymized electronic research data base, this study was approved by the center’s IRB based on expedited review.

## Results

**Fig. 1:**
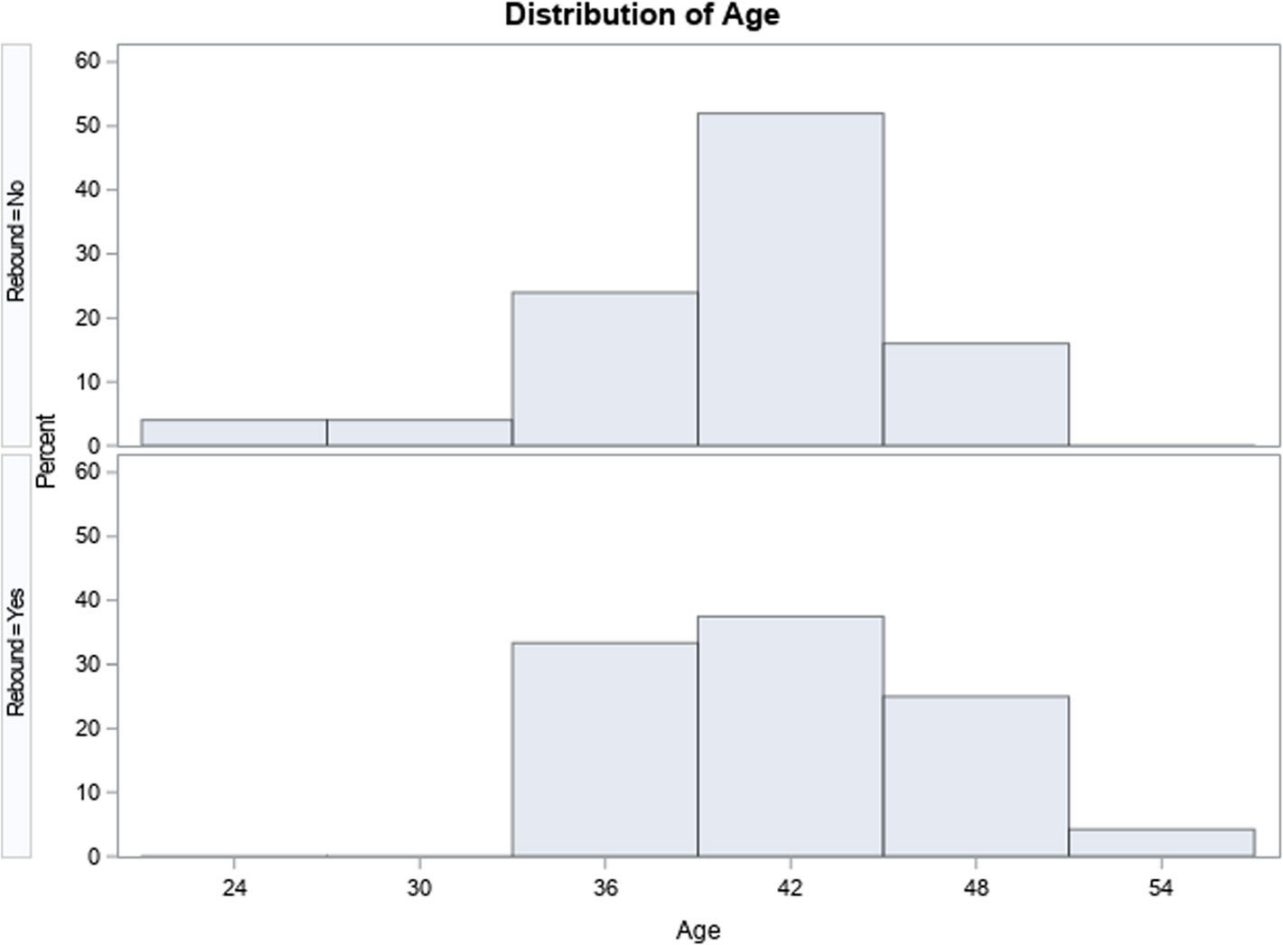
Age distributing of no-rebound and yes-rebound patients. Counterintuitively, no-rebound patients included younger women < age 33, while yes-rebound patients extended into the 50s.

This study involved 49 women with mean age 40.5 ± 5.1 years (range 23–52). Though women with and without rebound did not differ significantly in median ages (no rebound 40.0 ± 6.0; rebound 41.0 ± 7.0 years, *P* = 0.41), Fig. 1 demonstrates somewhat unexpected differences in age distribution, with women who failed to rebound including more younger women (< age 33 years), while women who did rebound extending into their 50 s.

Among those 49 women, 25 recorded no rebound effect (51.0%) and 24 did (49.0%). Table 1 presents a comparison between patients with and without rebound effects. As the table demonstrates, the differences between both groups were small: There was no significant difference in age, highest FSH recorded, last FSH recorded before cycle start, first E_2_ at presentation and E_2_ at initial stimulation withdrawal. Statistically significant differences were only found in AMH levels (*P* = 0.0026) and last E_2_ (*P* < 0.01) after reinstatement of exogenous stimulation and before hCG trigger. The statistically significant difference in AMH appears, however, clinically irrelevant since levels in both groups were extremely low, while the significant difference in last E_2_, of course, is a reflection of successful stimulation of follicles in patients who experienced a rebound.

**Table 1:** Patient characteristics of patients with and without rebound effect

	NO rebound *n*=25	YES rebound *n*=24	*P*-value
Age (years)	40.0± 6.0	41.0±7.0	0.41 n.s.
Max FSH (mIUmL)	40.0±58.7	20.9±20.8	0.07 n.s.
Last FSH^a^ (mIU/mL)	20.7±51.7	13.3±13.0	0.20 n.s.
AMH (ng/mL)	0.0±0.1	0.1±0.2	0.003
1st E2 (pg/mL)^a^	41.9±21.2	41.4±19.8	0.77 n.s.
Last E2 (pg/mL)^b^	45.2±19.4	162.0±70.1	<0.01
E2 at rebound start (pg/mL)	46.7±32.8	55.5±24.1	0.43 n.s.

^a^Last measurement before IVF cycle start (i.e., after at least 6 weeks of DHEA and CoQ10supplementation)

^b^Last measurement before hCG trigger.

Among 24 women with positive rebound, 1–3 oocytes were retrieved in 21 (87.5%) women, representing 42.9% of all patients who underwent a rebound attempt. Among those 21 patients, in 14 (58.3% and 28.6%, respectively) 1 oocyte was retrieved, in 4 (16.7% and 8.2%) 2 oocytes and in 3 (12.5% and 6.1%) 3 oocytes. Moreover 13 patients (54.2% among those with rebound and 26.5% of the total study population) fertilized 1 oocyte and 4 (16.7% and 8.2%, respectively) fertilized 2 oocytes.

A total of 15 patients reached embryo transfer (30.6% of all patients), 11 (22.4%) with single embryo and 4 women with 2 embryos (8,2%). At time of this report, an ongoing clinical pregnancy has not yet been established.

Of here reported patients, 3 had subsequent rebound cycles, meaning that they in subsequent cycles again did not respond to initial exogenous stimulation. Among those, 2 had one additional cycle and failed to rebound; 1 patient had 2 additional rebound cycles in which she produced 1 and 2 oocytes, respectively, but none fertilized.

## Discussion

Results of here presented study at several levels were unexpected: A first was the recognition that approximately half of all study participants (i.e., complete non-responders) rebounded within a few short days of interruption in ovarian hyperstimulation and, on their own, developed growing follicles without exogenous gonadotropin support. While we had observed such rebounds anecdotally before, our clinical impression had been that successful rebounds were much rarer, possibly somewhere around ca. 15%. Recording in this prospective study more than triple the expected rate, was highly unexpected and must be assumed in the long-term to lead to additional pregnancies. That these rebounds, however, only represented last vestiges of ovarian function is supported by the poor cycle outcomes in those three women who wanted to repeat successful rebounds leading but failed.

A second unexpected finding was the observation that, at least at initial stages, these follicles, apparently, had enough endogenous hormonal support to sustain growth without help from exogenous gonadotropins. This conclusion is supported by the in this study used definition of a successful rebound, - autonomous follicle growth and increased E_2_ production over 48 hours, following initial identification of a growing follicle with vaginal ultrasound and rising E_2_. Our earlier experience with rebounds, where in years we were unable to achieve a successful rebound after more than 7 days of unsuccessful stimulation, precludes the possibility that these rebounds only represent a late response to stimulation. Where this support stems from, has been subject of many of our center’s clinical case reviews and discussions at research meetings.

The study’s design also speaks against another possible explanation, namely that the initial high-dosage gonadotropin stimulation desensitized the pituitary to gonadotropin levels. This hypothesis appears rebutted by the observation that reinitiating gonadotropin stimulation does not require maximum stimulation dosages our center usually initiates stimulation in poor-prognosis patients with LFOR (450–600 IU of FSH and hMG daily), as the study protocol only utilized 225–300 IU of hMG in exogenous support of growing follicles.

A third unexpected finding, considering ages and poor ovarian reserves of patients in this study, was the observation that among women with rebound response, up to 3 oocytes were retrieved, which in the long run should result in pregnancies, even though in the first 15 transfers no ongoing clinical pregnancy has been established. Morphologically, we were unable to detect differences in oocytes and embryos generated in rebound cycles from normally progressing stimulation cycles. Considering here reported patient population, low pregnancy chances are, however, fully expected [1]. Even women who demonstrated a rebound had a median age of 41.0 ± 7.0 years, a maximal FSH of 20.9 ± 20.8 mIU/mL and AMH of only 0.1 ± 0.2 ng/mL, all highly adverse prognostic parameters predictive of extremely poor pregnancy chances.

After age, the number of embryos available for transfer in such patients is the second-best predictor of pregnancy and live birth success [5]. The relatively small number of embryos available for transfer in these patients also reemphasizes their low probability of conception. But even small chances of pregnancy and live birth in such poor prognosis patients have value [1] and, actually, represent an argument for further research pursuits involving use of rebounds in very poor prognosis patients.

A somewhat disappointing finding of the study was that it did not reveal a patient phenotype predictive of successful rebounds. The similarity between rebounding and non-rebounding patients was, indeed, quite remarkable (Table 1). Moreover, even though ages between the groups did not differ, remarkably, the rebounding group of patients included women into their 50 s, while the non-rebounding group included some women under age 33 (Fig. 1). Rebounds, therefore, should be attempted in every patient who is non-responsive to stimulation before such patients’ cycles are cancelled, which usually means a referral into third-party egg donation or experimental treatment protocols. As this study demonstrates, at our center this clinical approach includes women up into their early 50 s, even though our center’s so-far oldest patient who delivered a child with use of autologous oocytes, was two weeks short of her 48th birthday at time of embryo transfer [1].

Additional efforts, therefore, must be directed at improving pregnancy and live birth chances with autologous oocytes for poor prognosis patients but, even more importantly, an understanding of the physiology of here reported rebounds is urgently needed [6]. Considering that roughly half of all patients responded with a rebound after demonstrating zero response to maximal exogenous stimulation with gonadotropins, this spontaneous response must be dependent on a specific endogenous physiological process. What that may entail, can as of this point only be hypothesized. Considering on various additional options we, as above noted, dismissed, we settled on the following hypothesis that, of course, requires confirmation: Sudden cessation of exogenous high-dose gonadotropin stimulation triggers an endogenous rebound of gonadotropin production by the pituitary with better “fitting” isoforms and/or glycosylation profiles of FSH and luteinizing hormone (LH) for poorly responding patients than exogenous gonadotropins can offer.

That isoform mixtures of glycoprotein hormones, like FSH, change during the menstrual cycle and with advancing age, has been known for some time [7]. Glycoforms of these hormones exist in large numbers and varieties of isoforms, characterized by their glycan content of terminal anionic monosaccharides, namely sialic acid and sulfonated N-acetylgalactosamine [8]. Endogenous natural ovarian stimulation appears driven by FSHtri and FSHtetra (3 and 4 N-glycans, respectively) as well as LHdi and LHtri (2 and 3 N-glycans, respectively) [9]. Likely due to greater receptor-binding affinity and occupation of more FSH binding sites [10], hypoglycosylated FSH^21/18^ appears to be clinically significantly more effective than fully glycosylated FSH, as present in glycosylated recombinant FSH^24^ [11]. Age-associated reduction in hypoglycosylated FSH^21/18^ further reduces the biological activity of circulating levels of FSH, potentially further compromising ovarian function [10]; yet, may still retain enough activity to stimulate an older ovary in a rebound scenario.

No other explanation than above outlined hypothesis, as of this moment, can explain here observed findings following sudden withdrawal of exogenous stimulation with fully glycosylated FSH in mostly older women with LFOR and complete ovarian resistance to exogenous stimulation. Either mediated by sudden availability of FSH receptors on granulosa cells to endogenous FSH isotypes after withdrawal of exogenous gonadotropins, or a possible negative feed-back mechanism between sudden exogenous gonadotropin withdrawal and GnRH releasing hormone in the hypothalamus, leading to pituitary stimulation of endogenous FSH production, currently appear to be the only possible explanations for the observed remarkably high rebound rate. Assuming this, indeed, to be the mechanism by which rebounds are achieved in such highly unfavorable patients, one must wonder whether these effects cannot also be harnessed in better prognosis patients.

If confirmed, several potential applications come to mind, including intermittent, in place of continues, ovarian stimulation with exogenous gonadotropins to co-op endogenous gonadotropin activity, thereby potentially saving medication costs. Most importantly, however, these possibilities raise the option of age-specific ovarian stimulations with exogenous gonadotropins that match patient-ages, rather than, as is currently the practice, treating all patients, independent of age, with exactly the same combination of gonadotropin isotypes. Orvieto and Seifer recently expressed similar ideas when, in discussing biosimilar FSH preparations, making the point that dose-response curves should be establishes for new exogenous biosimilar gonadotropins in well-defined patient populations [12].

It also would seem illogical from an evolutionary viewpoint for nature to change the isotype profile of FSH within cycles and with advancing age, unless there was a physiological purpose to such changes. One seemingly obvious purpose in advancing age would be that FSH isotypes have to adjust to changing demands of aging granulosa cells. This does not necessarily mean that aging granulosa cells are in need of more effective (i.e., more hypo-glycosylated) isotypes, as has been suggested [11]; they could, simply, be in need of more “compatible” isotypes with older granulosa cells. On the other hand, one can also make a case for using more hypo-glycosylated FSH isoforms in older women and other poorer-prognosis patients in order to drive ovaries harder [11].

## Conclusions

Measuring FSH isotypes in peripheral blood is, unfortunately, complex and only very few research laboratories have the ability [8, 13]. Identification of specific FSH isotypes responsible for here reported rebounds could facilitate production of age-specific exogenous gonadotropins in place of uniform gonadotropin products that are currently marketed for use in women of all ages. It has been known for some time that the endogenously produced isotypes of FSH change as women age [11]. That exogenous gonadotropin therapy, therefore, should change in parallel appears obvious, - yet, surprisingly, has not been explored by the pharma industry. Hopefully, this study may offer some new incentives.

## Figures and Tables

**Fig. 2:**
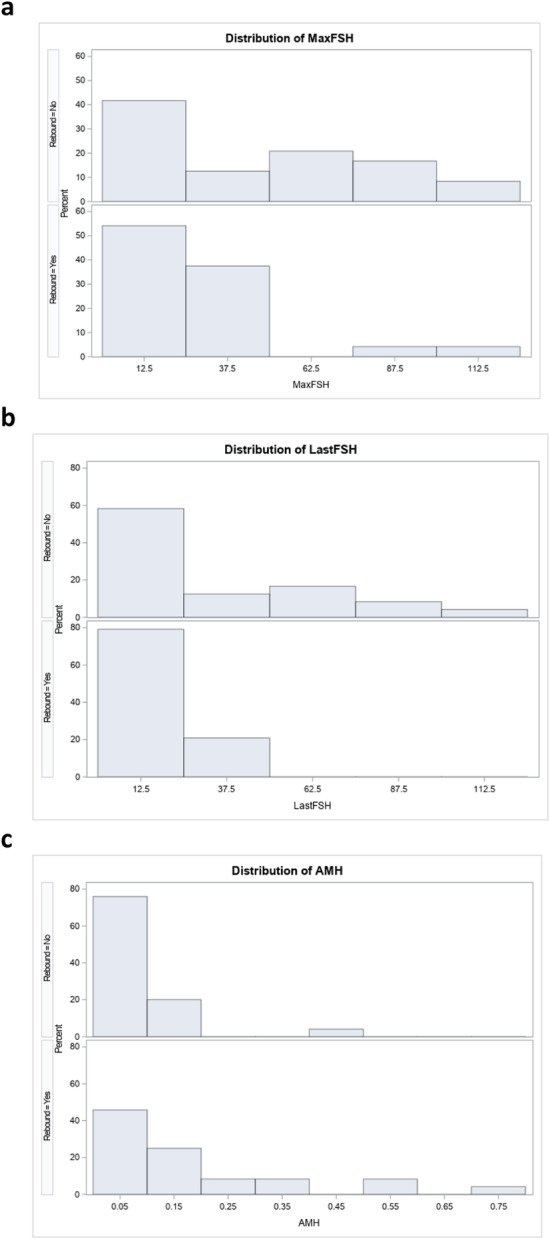
Non-parametrical distributions of (**a**) highest FSH; (**b**) last FSH; (**c**) AMH

**Fig. 3:**
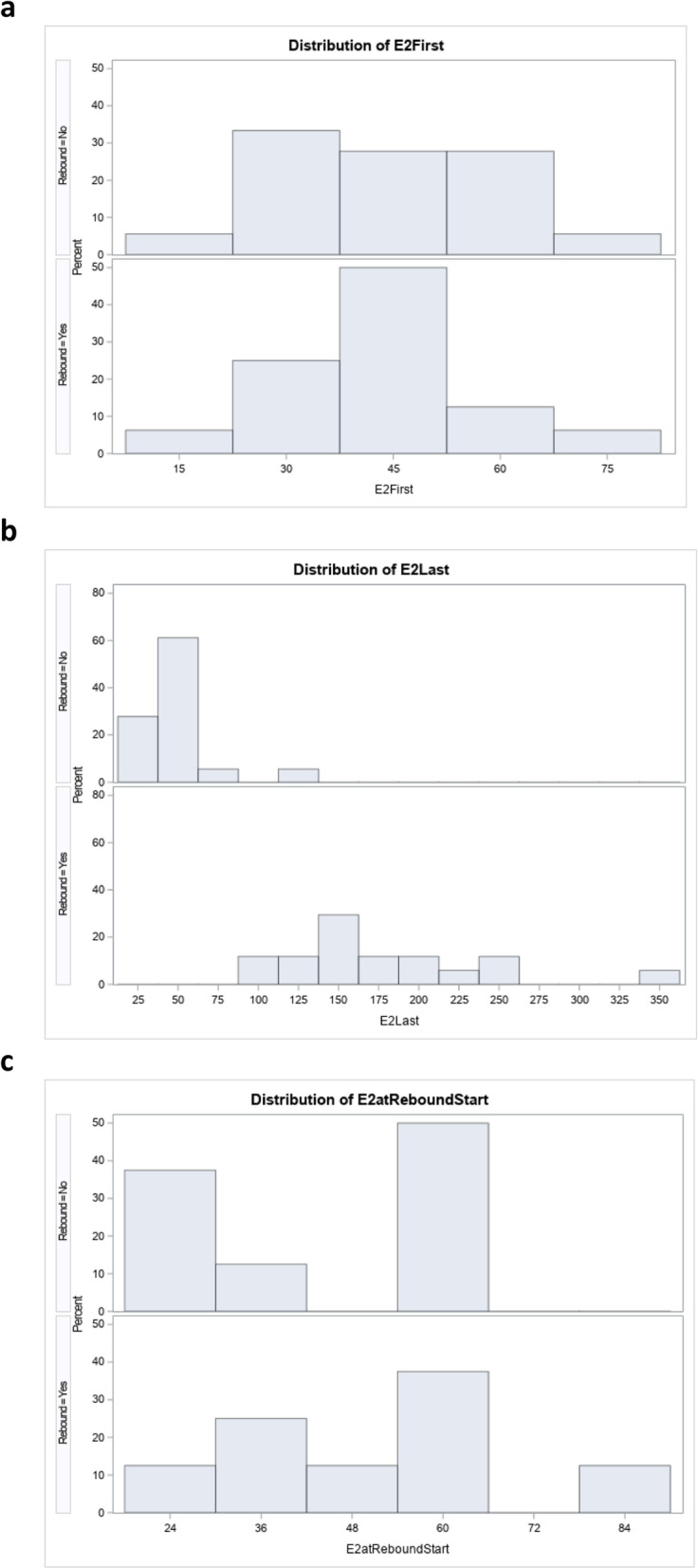
Distribution (**a**) first E2; (**b**) last E2 and (**c**) E2 at rebound start

## Data Availability

All data and material are contained in the manuscript.

## References

[1] Albertini DF, Barad DH, Darmon S, Gleicher N, Kushnir VA. Older women using their own eggs? Issue framed with two oldest reported IVF pregnancies and a live birth. Reprod Biomed Online. 2018 Aug;37(2):172–7. 10.1016/j.rbmo.2018.05.010 29936089

[2] Barad DH, Gleicher N, Weghofer A (2007). Age-specific levels for basal follicle-stimulating hormone assessment of ovarian function. Obstet Gynecol.

[3] Barad DH, Gleicher N, Weghofer A (2011). Utility of age-specific serum anti-Müllerian. hormone concentrations Reprod Biomed Online.

[4] Albertini DF, Barad DH, Darmon SK, Gleicher N, Kushnir VA, Wang Q, Zhang L, Wu YG (2018). With low ovarian reserve, Highly Individualized Egg Retrieval (HIER) improves IVF results by avoiding premature luteinization. J Ovarian Res..

[5] Albertini DF, Barad DH, Darmon SK, Gleicher N, Kushnir VA, Sen A, Wang Q, Weghofer A, Wu YG, Zhang L. Definition by FSH, AMH and embryo numbers of good-, intermediate- and poor-prognosis patients suggests previously unknown IVF outcome-determining factor associated with AMH. J Transl Med. 2016 Jun;10(1):172. 14(. 10.1186/s12967-016-0924-7 PMC4901433 27286817

[6] Adashi EY, Barad DH, Gleicher N (2020). Why is use of donor eggs not viewed as treatment failure? A call for improvements in treatment with autologous oocytes. J Assist Reprod Genet.

[7] Yding Andersen C. Effect of FSH and its different isoforms on maturation of oocytes from pre-ovulatory follicles. Reprod Biomed Online 202;5(3):232–239. 10.1016/s1472-6483(10)61826-3 12470520

[8] Eriksson K, Wide L (2017). Molecular size and charge as dimensions to identify and characterize circulating glycoforms of human FSH, LH and TSH. Ups J Med Sci.

[9] Eriksson K, Wide L (2018). Low-glycosylated forms of both FSH and LH play major roles in the natural ovarian stimulation. Ups J Med.

[10] Butnev VY, Butnev VY, May JV, Shuai B, Tran P, White WK, Brown A, Smalter Hall A, Harvey DJ, Bousfied GR (2015). Production, purification, and characterization of recombinant hFSH glycoforms for functional studies. Mol Cell Endocrinol.

[11] Bousfield GR, Butnev VY, Davis JS, Jiang C, Hou X, May JV, Wang C (2015). Hypoglycosilated hFSH has greater bioactivity than fully glycosylated recombinant hFSH in human granulosa cells. J Clin Endocrinol Metab.

[12] Seifer DB, Orvieto R (2016). Biosimilar FSH preparations- are they identical twins or just siblings?. Reprod Biol Endocrinol.

[13] Aguzzoli L, Casarini L, Daolio J, De Pascali F, Galano E, Klett D, Lazzaretti C, Limoncella S, Nicoli A, Palmese Am Satwekar A, Paradiso E, Poti F, Reiter E, Riccetti L, Simoni M, Sperduti S, Tagliavini S, Trenti T, Villani MT (2019). Glycosylation pattern and *in vitro* bioactivity of reference follitropin alfa and biosimilars. Frontiers Endocrinol.

